# Poly(styrene-*co*-maleic acid) Micelle of Photosensitizers for Targeted Photodynamic Therapy, Exhibits Prolonged Singlet Oxygen Generating Capacity and Superior Intracellular Uptake

**DOI:** 10.3390/jpm12030493

**Published:** 2022-03-18

**Authors:** Gahininath Yadavrao Bharate, Haibo Qin, Jun Fang

**Affiliations:** 1 Laboratory of Microbiology and Oncology, Faculty of Pharmaceutical Sciences, Sojo University, Kumamoto 860-0082, Japan; gahininath@gmail.com (G.Y.B.); heaurt@hotmail.com (H.Q.); 2 Department of Applied Chemistry, Graduate School of Engineering, Sojo University, Kumamoto 860-0082, Japan; 3 SKE Labs, Survery No. 7/1, Jai Malhar Nagar, Thergaon, Pune 411033, India; 4 Department of Applied Microbial Technology, Graduate School of Engineering, Sojo University, Kumamoto 860-0082, Japan

**Keywords:** polymeric micelles, macromolecular photosensitizers, targeted drug delivery, SMA-micelles, singlet oxygen

## Abstract

Targeted therapy by using nanomedicines based on the enhanced permeability and retention (EPR) effect is becoming a promising anticancer strategy. Many nano-designed photosensitizers (PSs) for photodynamic therapy (PDT) have been developed which show superior therapeutic potentials than free PS. To further understand the advantages of nano-designed PS, in this study, we used styrene-co-maleyl telomer (SMA) as a polymer platform to prepare a micellar type of PS with two well-characterized PSs—rose bengal (RB) and methylene blue (MB)—and evaluated the outmatching benefits of SMA-PS micelles, especially focusing on the singlet oxygen (^1^O_2_) generation capacity and intracellular uptake profiles. In aqueous solutions, SMA-PS self-assembles to form micelles by non-covalent interactions between PS and SMA. SMA-PS micelles showed discrete distributions by dynamic light scattering having a mean particle size of 18–30 nm depending on the types of SMA and different PSs. The hydrodynamic size of SMA-PS was evaluated by Sephadex chromatography and it found to be 30–50 kDa. In the presence of human serum albumin, the sizes of SMA-PS remarkably increased, suggesting the albumin-binding property. ^1^O_2_ generation from the SMA-PS micelle was determined by electron spin resonance, in which the SMA-PS micelle showed comparatively more photo-stable, and consequently a more durable and constant, ^1^O_2_ generation capability than free PS. Moreover, intracellular uptake of SMA-PS micelles was extensively faster and higher than free PS, especially in tumor cells. Taken together, SMA-PS micelles appear highly advantageous for photodynamic therapy in addition to its capacity in utilizing the EPR effect for tumor targeted delivery.

## 1. Introduction

Treatment of diseases using light irradiation has had a long history since Finson’s work on photodynamic therapy (PDT) for cutaneous tuberculosis via generation of singlet oxygen (^1^O_2_), which resulted in a Nobel Prize in 1903 [1,2]. Difficulties do exist in treating deep tumors by PDT, because the optical properties of tissues offer limited light penetration. However, successful applications of PDT have been developed and obtained approval for clinical use, especially for treating superficial tumors and for some other tumors where the endoscopic light source is accessible, for example, esophageal epithelial cancer [3,4,5]. Although many photosensitizers (PSs) such as photofrin, levulan, foscan, metvix and leserphyrin are commercially available for clinical use [6], these are low molecular weight agents which have crucial shortcomings when they are injected intravenously. Namely, these are distributed or accumulated to all the organs or tissues indiscriminately, and only a negligibly small fraction reaches the desired tumor tissues [7,8,9]. Indiscriminate tissue distribution of PSs may exert adverse effects. The accumulation of the PS to the skin is a major concern, as skin irritation or damage occurs frequently after PDT even under ambient light exposure [6,7]. Patients receiving PDT using conventional low molecular weight PS are usually photosensitive for several days to weeks, and thus, it is recommended for the patients to avoid excess ambient light [6].

In this context, macromolecular PS for PDT will outperform over free PS of low molecular weight because those will be delivered to tumor tissues more selectively based on the enhanced permeability and retention (EPR) effect [10,11,12,13,14]. The EPR effect is a unique pathophysiological phenomenon regarding vascular permeability in the solid tumors, namely, compared to normal tissues, tumor tissues show abnormal vasculature with large gaps between endothelial cells of blood vessels and high vascular permeability, as well as defected lymphatic functions [10,11,12,13]. Consequently, biocompatible high molecular weight drugs with molecular weights higher than 40–50 kDa, or molecular sizes larger than 5–10 nm, would accumulate more selectively in the tumor, and with only a marginal distribution in normal tissues; for example, the tumor concentration of macromolecular drugs, in relation to that of blood, can be 10- to 20-fold higher [10,11,12,13]. Furthermore, prolonged circulation time and retention time in the tumor, for instance, several days in mice, will be attained in vivo by using macromolecular drugs or nanomedicines. In contrast, low molecular weight drugs disappear in a few minutes or hours [11,12,13]. Thus, the prolonged circulatory property of macromolecular drugs is a big advantage over its low molecular weight counterpart. Consequently, more effective PDT, while an avoiding adverse effect, could be achieved by using nano-designed PSs. To date, tumor targeted PDT has been developed by using various macromolecular formulations of PSs with sizes of several to several hundred nanometers, in which biocompatible polymers, liposomes, and antibodies are utilized to modify PSs [15,16,17]. In line with the PDT principle, recently, redox-triggered light-independent therapy, i.e., chemodynamic therapy by using silica nanoparticles of carbon dots and copper, has been developed which exhibits promising potential for cancer theranostics [18,19]. In our laboratory, by using biocompatible polymers of polyethylene glycol (PEG), styrene maleic acid copolymer (SMA), and poly(N-(2-hydroxypropyl) methacrylamide) copolymer (HPMA), we have developed several polymeric micellar PSs including PEG conjugated zinc protoporphyrin (ZnPP) [20,21], SMA micelles of ZnPP and temoporfin [7,21,22,23], HPMA conjugated ZnPP [24,25], and HPMA conjugated pyropheophorbide-a [26], all of which showed superior PDT effect and safety profiles benefiting from their tumor-targeting properties.

Along this line, in this study, we aimed to develop SMA micelles of rose bengal (RB) and methylene blue (MB) for tumor-targeted PDT. Both RB and MB are phenothiazine dyes and well-characterized PSs [27]. MB has already been approved and is being applied in the clinical treatment regimen of caries and periodontal diseases. For MB-based PDT, red light of 660 nm is applied as a light source for irradiation, which is close to the near-infrared window, and thus exhibits deep tissue penetration properties [27]. The potentials of RB as a PDT are relatively less explored. Though RB has not been applied in a clinical setting, RB combined with a green laser source was proposed as a more efficient anti-bacteria PDT candidate than MB [17]. RB is widely available, biocompatible, cheap, and it is used as a food coloring agent or pigment in Japan [27]. Though the applications of these two PSs have been firstly focusing on anti-microbial PDT [27], many studies have revealed their applicability in anticancer PDT, both in vitro and in vivo [28,29,30,31]. Regarding tumor-targeted nano formulations of RB and MB, various nano-systems have been developed recently, for example, Wu et al. reported a liposomal formation of MB which showed a potent PDT effect against breast cancer cells [28]; Jesus et al. developed gold nanoparticles and silver nanoparticles of MB which exhibited significantly higher cytotoxicity than free MB against human breast cancer cells [30]. 

The polymeric micelle is a well-accepted nano-platform where self-assembly of amphiphilic polymers in aqueous solutions encapsulates small molecular drugs in the core of micelles [32,33]. SMA is an example of an amphiphilic copolymer which has a hydrophobic styrene motif and a hydrophilic maleic acid motif. We have successfully developed several SMA micelles of anticancer agents including doxorubicin, pirarubicin, and ZnPP, all of which showed stable micelle structures in physiological aqueous solutions and in circulation, and the albumin-binding property of SMA further renders prolonged circulation time and improved stability of SMA micelles, consequently achieving tumor-targeted therapeutic effects [22,34,35]. Ideal micellar drugs, as well as other nanomedicine for targeted anticancer therapy, should be stable in circulation for a reasonable period of time to ensure sufficient EPR effect-based tumor accumulation, whereas they should also release active drugs in tumor tissues and/or be taken up by tumor cells rapidly, to achieve satisfactory therapeutic effect [13]. In addition, for targeted PDT using nano-designed PSs, beyond the EPR effect-based tumor targeting, the capacity and duration of ^1^O_2_ generation is also a critical issue to affect the therapeutic outcome. However, this issue of the stability and capacity of nano-designed PS to generate ^1^O_2_ has not been extensively investigated yet. In this context, aiming to further reveal the potential and advantages of the SMA micelle of PS for anticancer PDT, by utilizing the two well-characterized PSs (i.e., RB and MB), we report the preparation, characterization and evaluation of micellar PS using SMA—i.e., SMA-RB and SMA-MB—especially focusing on their ^1^O_2_ generation profiles upon light irradiation, the stability of ^1^O_2_ producing capacity, and their cellular uptake properties.

## 2. Materials and Methods

### 2.1. Materials

Partially (about 37%) half-butyl esterified SMA (buSMA) and maleyl dicarboxy SMA (cSMA), both as the maleyl anhydride form of SMA, as described by Maeda et al. [36], were obtained from Kuraray Co. Ltd., Kurashiki, Japan. RB, MB, 2,2,6,6-tetramethyl piperidine (TMP), and all other reagents of reagent grade were purchased from Wako Pure Chemical Industries Ltd., Osaka, and used without further purification.

### 2.2. Preparation of SMA-PS Micelle 

Two types of SMA, i.e., cSMA with mean molecular weight (Mw.) of 1360 and buSMA with mean Mw. of 1580, were used to prepare SMA-micelles. The maleic anhydride groups of both SMA were hydrolyzed using 0.1 M NaOH at 60–70 °C for 24 to 30 h, and the hydrolysates were further purified by differential precipitation at pH 3.0 using 0.1 M HCl. The SMA-PS micelle was prepared by using the protocol as described before in our laboratory for SMA micelles [22,34,35] with modifications. Briefly, hydrolyzed SMA (180 mg) was dissolved in deionized water (36.0 mL) under stirring, and the pH of the SMA solution was increased to 9.5 by adding 0.1 M NaOH. Then, 20 mg of PS dissolved in 20 mL of deionized water was added dropwise under stirring. This solution mixture was stirred for 3.0 h at room temperature, and then the pH was brought down to 3.0 by dropwise addition of 0.1 M HCl to precipitate the SMA-PS micelles. The precipitate was then centrifuged (8000 rpm × 10 min) and washed twice with 100 mL of cold HCl (0.01 M). The precipitates were then suspended in 50 mL of deionized water and the pH was then adjusted to 7.4 by dropwise addition of 0.1 M NaOH. Finally, the micelle was subjected to ultrafiltration using the Millipore Lab Scale TFF system (Millipore, Bedford, MA, USA) with a membrane cut-off size of 10 kDa, then lyophilized to obtain fluffy SMA-PS micelles. The yields of all SMA-PS micelles were shown in Table 1.

### 2.3. Characterization of SMA-PSs Micelles

#### 2.3.1. Size Exclusion Chromatography

Size exclusion chromatography of each SMA-PSs micelle in aqueous medium was carried out with Sephadex G-100 superfine (Amersham Biosciences AB, Uppsala, Sweden) in a column (size: Ø1.5 cm × 87 cm) by using 0.2 M sodium phosphate buffer (pH 7.4) as eluent. Each 3.2 mL eluted fraction was monitored by absorption at 549.5 nm for SMA-RB, 664 nm for SMA-MB, and 260 nm for SMA, respectively. The peak fraction was pooled, dialyzed against deionized water, and lyophilized. The lyophilized powder thus obtained was subjected to further characterization by absorption and fluorescence spectroscopy. To estimate the apparent molecular size of these micelles, the peak positions were compared with various standard proteins, i.e., immunoglobulin (IgG; 155 kDa), bovine serum albumin (BSA; 67 kDa), D-amino acid oxidase (DAO; 38 kDa), and neocarzinostatin (NCS; 12 kDa). 

#### 2.3.2. UV-Visible and Fluorescence Spectroscopy

UV/visible absorption spectra were obtained by the UV/Vis spectrophotometer Model U-3900 (Hitachi, Tokyo, Japan). The loading of PS in SMA-PS micelles was quantified using free PS standard absorption at λmax 549.5 nm and 664.0 nm for SMA-RB and SMA-MB, in 0.2 M sodium phosphate buffer (pH 7.4), respectively. 

Fluorescence spectra were obtained by using spectrofluorometer Model FP-6600 (Jasco, Tokyo, Japan). The sample solution of SMA-PS micelles was excited at 520.0 nm for RB and 630.0 nm for MB, respectively. The emissions from 540 to 700 nm for SMA-RB and 650.0 nm to 750 nm for SMA-MB were measured. 

#### 2.3.3. Particle Size Analyses by Dynamic and Static Light Scattering, and the Zeta Potential for Surface Charge Determination

Particle size distribution was measured by using the Photal Model DLS-7000 HLs laser-light scattering spectrophotometer (Otsuka Electronics Inc., Osaka, Japan), equipped with a He-Ne (632.5 nm) laser light source. For DLS measurements, the scattering angle was fixed at 90° and the temperatures of the samples were maintained at 25.0 ± 0.1 °C. Each sample concentration was at 1.25 mg/mL prepared in 0.2 M sodium phosphate buffer (pH 7.4). 

Static light scattering (SLS) measurements were carried out at 25 ± 0.1 °C to obtain molecular weights of each SMA-PS. The DLS-7000 instrument (Otsuka Electronics Inc.) that is equipped with Zimm, Zimm Square, Berry, and Debye plots software for the analysis of static light scattering data was used. SLS measurements of SMA-PS solution were carried out in 0.2 M phosphate buffer (pH 7.4) at a concentration ranging from 0.2 mg/mL to 2.5 mg/mL, and samples were analyzed by at least seven different angles (θ), ranging from 30° to 140°. The method of the Zimm Sq. plot was used to obtain molecular weight (Mz). 

The surface charge of the SMA-PS micelles was determined by zeta potential and a particle size analyzer (Photal Model ELSZ-2, Otsuka Electronics, Osaka, Japan) at room temperature in deionized water. The concentrations of the SMA-PS at 1.25 mg/mL were used for the zeta potential measurement.

#### 2.3.4. Transmission Eletron Microscopy (TEM)

The shape and size of SMA-PS were also determined by TEM. cSMA-RB and cSMA-MB were dissolved in deionized water at 5 mg/mL, and 50 μL of the solution were mixed with 50 μL of 0.01% phosphotungstic acid, after which the mixture was transferred to a grid for TEM analysis at above 80 kV (JEM-1400 plus; JEOL, Tokyo, Japan).

### 2.4. Binding of SMA-PS Micelle to Human Serum Albumin

Sephacryl-S200 HR (Sigma) gel chromatography of SMA-PS with or without human serum albumin (HSA) was carried out to determine apparent molecular size of SMA-PS micelles in an aqueous system, and examined the property of SMA for albumin binding. In brief, SMA-RB (2.0 mg/mL) was first dissolved in 0.2 M sodium phosphate buffer pH 7.4. Then, different amounts of HSA (0–10 mg/mL) were added and incubated for 30 min at room temperature. Then, 1.0 mL of each sample was subjected to a Sephacryl-S200 HR column (size: 1.5 cm Ø × 48 cm (h)), and eluted with 0.2 M sodium phosphate buffer (pH 7.4); a 3.2 mL fraction was collected from each tube, being monitored at 280 nm for HSA and 549 nm for RB micelles.

### 2.5. Release Rate of Free PS from the SMA-PS Micelles

Rates of free PS release from the SMA-PS micelles were determined by absorbance of dialysate. Each micellar solution (1.0 mL; 1.0 mg/mL) was placed in dialysis tube (Spectrapor, Spectrum Laboratories Inc., San Diego, CA, USA) with Mw cut off of 10 kDa in 0.2 M phosphate buffers (35.0 mL each) of pH 6.0, 7.5 and 9.0 under shaking at 1 Hz in a water bath at 37 °C. The release of free RB from SMA-RB and MB from SMA-MB at the outside of the dialysis tube were quantified by absorption at 549.5 and 664.0 nm, respectively. 

### 2.6. Electron Spin Resonance (ESR) Spectroscopy: Quantification of Singlet Oxygen Generation

Electron spin resonance (ESR) spectra were obtained at 25 °C by using a model JES FA-100 Spectrometer (JEOL, Tokyo, Japan). Sample solutions containing 0.5 µM SMA-PS or free PS and 30 mM of TMP with or without light irradiation were measured. Samples in the quartz flat cell (Labotec, Tokyo, Japan) were irradiated multiple times repeatedly or by one shot. In another set of experiments, TMP was added in a time-lagged manner (0–20 min) by the xenon light (109,000 LUX) source of a slide projector with a broad spectrum covering the excitation wavelengths of both MB and RB; this was conducted using a Kodak Ektagraphic III (Kodak Ltd., Rocheter, NY, USA) equipped with a 300 W xenon lamp. The ESR spectrometer was set at a microwave power of 2.0 mW, an amplitude of 200 kHz field modulation, and a width of 0.1 mT.

### 2.7. Bleaching Effect of Free PS and SMA-PS by Light Irradiation

Stabilities of both SMA-RB and SMA-MB upon light irradiation were studied by measuring ^1^O_2_ generation in comparison with free PS over a time course. Bleaching and degradation of PS and SMA-PS micelles were monitored by UV absorption spectroscopy over a time course by light irradiation. 

Both SMA-PS micelles at a concentration of 0.08 µM (PS eqvt.) in 0.2 M sodium phosphate buffer (pH7.4) were irradiated by one shot or lagged time addition of TMP in the same system described in Section 3.6 under air cooling. The light intensity used was 109,000 LUX at the surface of the cuvette. 

### 2.8. Internalization of Free PS and SMA-PS in Tumor and Normal Cells

Human esophageal cancer cell line (KYSE-150) generously supplied by Dr. Hiroshi Kumimoto, Aichi Cancer Center, Nagoya, Japan, was cultured in DMEM medium (Invitrogen-Gibco, Grand Island, NY, USA), supplemented with 10% fetal bovine serum (FBS; JRH Biosciences, Lenexa, KS, USA). The human retinal pigment epithelium cell line ARPE-19 was obtained from the American Type Culture Collection (ATCC, Mantissa, VA, USA), and was cultured in DMEM/Ham’s F12 medium (Invitrogen-Gibco), supplemented with 10% FBS (JRH Biosciences). The cells were cultured at 5% CO_2_ at 37 °C.

For intracellular uptake study, the cells were plated in a 12-well plate (50,000/well). After 24 h pre-incubation, free PS or SMA-PS (20 µM, PS equivalent) was added. After the scheduled time, the medium was removed, and the cells were washed with PBS, and subsequently, cells were lysed with 100 µL of 10% TritonX-100^®^ and 900 µL of 4 M HCl in ethanol per well. The cell lysates were collected and then treated at 70 °C for 50 min. After centrifugation at 12,000 rpm for 10 min at room temperature, the supernatant (500 µL) was transferred to a test tube and diluted with PBS (1.5 mL) for fluorescence measurement. In a separate study, the cellular uptake of free RB and SMA-RB micelles in the KYSE-150 cells was studied by using Eclipse TE-2000 (Nikon, Japan) confocal laser microscopy.

### 2.9. In Vitro Cytotoxicity Assay

Human esophageal cancer KYSE-150 cells were used for evaluating the in vivo cytotoxicity of SMA-PS with/without light irradiation. Cells (3000/well) were seeded in 96-well plates. After overnight pre-incubation, SMA-PS (cSMA-RB and cSMA-MB), as well as free RB and MB, were added at different concentrations. Two hours later, the media were removed followed by washing twice with PBS, and fresh media were added into the plates. Then, light irradiation was carried out by a xenon light source (MAX-303; Asahi Spectra) at 400–700 nm for 30 min (12 mW/cm^2^, 21 J/cm^2^). After a further 24 h of culture, the MTT assay was carried out to quantify viable cells. 

### 2.10. Statistical Analyses

All data were expressed as means ± SD. Data were analyzed by using ANOVA followed by the Bonferroni correction. A difference was considered statistically significant when *p* < 0.05.

## 3. Results

### 3.1. Characterization of SMA Copolymer and SMA-PS Micelles

**Figure 1:**
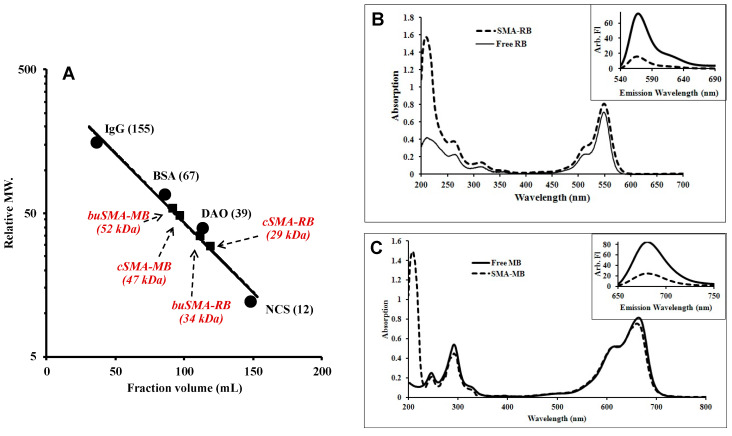
Sephadex superfine G-100 gel chromatography of SMA-PS micelle (**A**), and the absorption spectrums of free rose bengal (RB) vs. SMA-RB micelles (**B**) and free methylene blue (MB) vs. SMA-MB micelles (**C**). Size-exclusion chromatography of SMA-PS micelles was carried out with various known molecular weight proteins as a reference standard; a 3.2 mL fraction was collected per tube and 0.2 M sodium phosphate buffer (pH 7.4) was used as eluent. The absorption spectrums were recorded at 0.8 μg/mL concentration of Photosensitizers (PSs) equivalent on UV/vis in a 0.1 M phosphate buffer (pH 7.4). Insets of (**B**) and (**C**) show the fluorescence spectra of PSs and SMA-PS micelles.

Sephadex G-100 superfine high resolution gel chromatography revealed the apparent molecular weight of SMA-PS micelles based on the molecular weight of known proteins, as shown in Figure 1A. SMA-PS micelles exhibit Mw corresponding to 29 kDa and 47 kDa for cSMA-RB and cSMA-MB, respectively; whereas slightly higher molecular weights were observed with buSMA micelles, which were 34 kDa and 52 kDa, for buSMA-RB and buSMA-MB, respectively. Because the molecular weight of SMA used in this study wass about 1.5 kDa, these results indicate self-assembly of SMA-PS in aqueous milieu to form supramolecular micelles, similar to other polymer micelles reported earlier in our group [7,22,24,26,34,35,37]. The zeta potential of buSMA-PS (average range of −20 to −25 mV) micelles was lower than cSMA-PS (−43.0 to −50.0 mV) micelles because about 37% of carboxyl (COO-) groups were butylated in buSMA, resulting in the loss of a substantial amount of negative charge in SMA (Table 1). It also indicates that carboxyl groups are extending outside of the surface of micelles when dissolved in aqueous medium. The characterization of SMA-RB and SMA-MB micelles were summarized in Table 1.

The UV spectra of SMA-RB and SMA-MB are similar with free RB and MB, however, the fluorescence of SMA-RB and SMA-MB were largely quenched compared to free RB and MB (Figure 1B,C). These findings supported the micelle formation of SMA-PS in aqueous solution, which is similar to our previously reported SMA micelles [7,22,34,35]. 

### 3.2. Analysis of Particle Size Distribution and Molecular Weight Estimation of SMA-PS Micelles by Dynamic and Static Light Scattering

**Figure 2:**
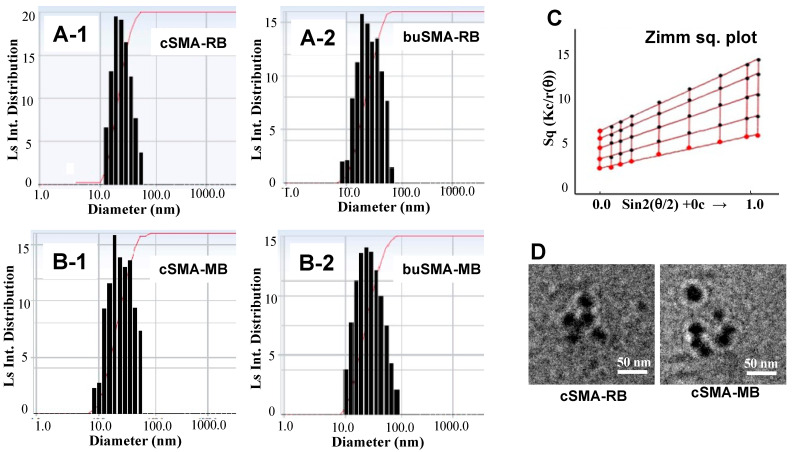
Dynamic light scattering of cSMA-RB (**A-1**), buSMA-RB (**A-2**), cSMA-MB (**B-1**) and buSMA-MB (**B-2**) micelles. Concentrations of each SMA-PS micelle were 1.25 mg/mL in 0.2 M sodium phosphate buffer (pH 7.4). The Zimm Sq. plot of cSMA-RB micelles by static laser light scattering is shown in (**C**). The particle size and shape were also measured by transmission electron microscopy (TEM) (**D**).

DLS studies of cSMA-RB and cSMA-MB in 0.2 M sodium phosphate buffer (pH 7.4) exhibited a mean particle size of 18.7 nm and 25.7 nm, respectively; whereas slightly larger molecular sizes were observed with buSMA-RB and buSMA-MB micelles, i.e., 23.5 nm and 27.5 nm, respectively (Figure 2A,B). However, in deionized water, lager particle sizes (e.g., 35.4 nm for csMA-RB) were observed compared to those in sodium phosphate buffer (Table 2).

Results of static light scattering (SLS) were shown in Figure 2C, in which the Zimm Square plot indicates a macromolecular complex being formed between PS and SMA telomer in aqueous medium. TEM also showed the spherical micelle formation of SMA-PS (Figure 2D). Hydrophobic interaction and hydrogen bonding between aromatic styrene rings and the maleyl side chain of SMA and PS molecules was expected to occur since amphiphilic detergents sodium dodecyl sulfate (SDS) and urea disrupted the micellar conformation, as depicted by the decrease of fluorescence quenching described below.

### 3.3. Albumin Binding of SMA-PS Micelles

**Figure 3:**
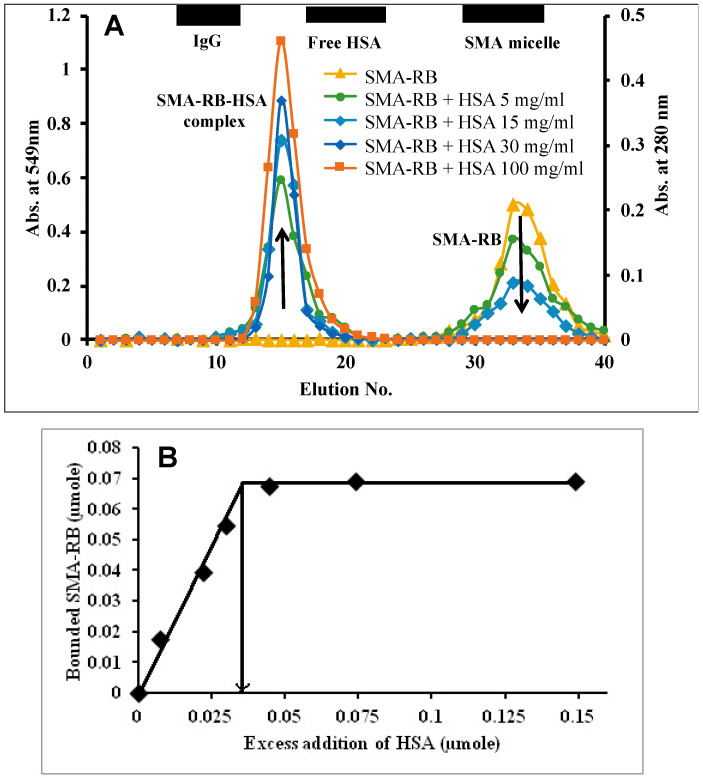
Size exclusion chromatography of SMA-RB in the presence and absence of Human serum albumin (HSA). (**A**) Gel chromatography of SMA-RB with/without HSA was performed using a Sephacryl S200 column (size: 1.5 cm Ø × 48 cm (L)) with the mobile phase of 0.2 M sodium phosphate buffer (pH 7.4). (**B**) HSA concentration vs. % bound SMA-RB curve for calculating the average amount of SMA-RB binding to HSA.

It is known that SMA micelles have an albumin binding property [22,34,35,38,39]. We thus anticipate that SMA-PS micelles will also show an increase in size after binding to serum albumin. To clarify this hypothesis, first Sephacryl-S200 gel chromatography of SMA-PS in the presence or absence of HSA was carried out. As shown in Figure 3, when different concentrations of HSA were added, the peak corresponding to SMA-PS decreased gradually in a dose-dependent manner. Accordingly, the peak of albumin-bound SMA-PS micelles was observed at the molecular size slightly larger than HSA (Figure 3A), suggesting the formation of an SMA-PS/HSA complex. DLS also showed an increase of micelle size in the presence of HSA (Table 2). Based on these results, we examined stoichiometry of albumin binding of SMA-PS micelles. Namely, to a given amount of SMA-RB micelle, an increasing excess amount of HSA was added to form a HSA-SMA-RB complex, and we found that 2.08 moles of SMA-RB bound per mole of albumin (Figure 3B).

### 3.4. Release of Free PS from SMA-PS Micelles

**Figure 4:**
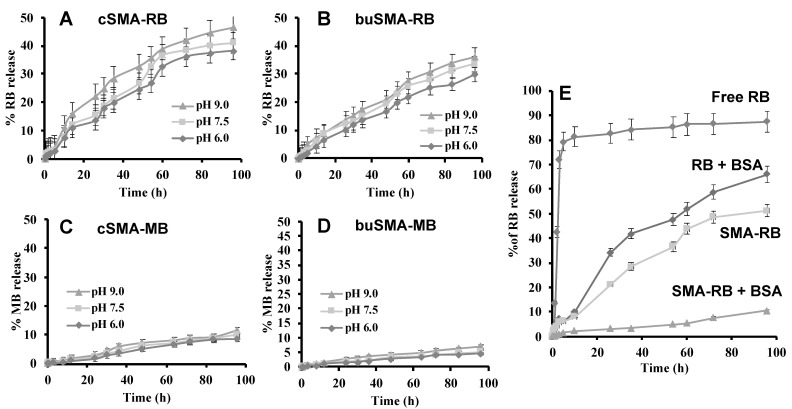
Release profiles of SMA-PS micelles; cSMA-RB (**A**), buSMA-RB (**B**), cSMA-MB (**C**), buSMA-MB (**D**), and SMA-RB micelles in the presence of bovine serum albumin (BSA) (**E**). SMA-PS micelles were dissolved in deionized water and sealed in a dialysis tube. At the indicated time after incubating at 37 °C, UV absorption at 549.0 nm and 664.0 nm corresponding to RB and MB was measured in the dialysate. Constant release rate from both cSMA-RB and buSMA-RB was observed at neutral pH. The release from cSMA-MB and buSMA-MB is considerably lower than cSMA-RB and buSMA-RB micelles (see text for details). Data are mean ± SD (*n* = 3).

Release of free RB and free MB from respective SMA micelles were examined up to four days in 0.2 M sodium phosphate buffers at different pHs at 37.0 °C. The results showed that release of free RB at pH 9.0 from the cSMA-RB was 10.0~11.0%/day at constant rate, which was slightly faster than that at pH 6.0 or 7.5 (both are 8.0~9.0%/day; Figure 4A). Release from buSMA micelles was slower than that of cSMA-RB micelles (7.5~8.0%/day) (Figure 4B), in phosphate buffer at 37.0 °C. Moreover, it is observed that release of free MB from SMA-MB micelles was much slower (2.0 to 2.5%/day) than SMA-RB (Figure 4C,D). This may be because of the positive charge on the sulfur atom and two dimethylamine residues in the structure of MB, which may have a strong ionic interaction with a negative charge on the carboxy group of the SMA chain. 

Furthermore, it was observed that release from SMA-RB micelles in the presence of BSA (2.0%) was very slow, i.e., 0.5–1.0% per day (Figure 4E), which is consistent with the results of binding of SMA-PS micelles to albumin, as shown in Figure 3. The slow release of PS in the presence of albumin from the micelles makes it advantageous to exhibit the EPR effect because it is known to take hours to exhibit the EPR effect-based tumor accumulation; i.e., PS micelles need to remain stable during circulation until it accumulates in the tumor [11,13].

### 3.5. Fluorescence Quenching as an Evidence of Tight Interaction of Aromatic Residues of PS to SMA in the Micelles and Destabilization of SMA-PS Micelle under Various Conditions

**Figure 5:**
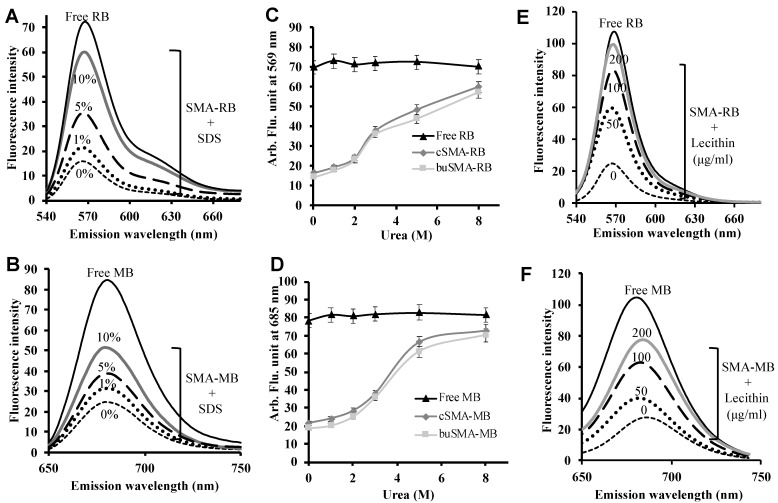
Fluorescence spectra of SMA-PS in the presence/absence of SDS (**A**,**B**), urea (**C**,**D**) and lecithin (**E**,**F**). The sample solutions were excited at 520.0 nm for RB and 630.0 nm for MB. Fluorescence quenching was showed as the much lower fluorescence intensity compared to free PS, and recovery of fluorescence quenching as shown by the increased fluorescence intensity indicates the disruption of micelle formation. Data are mean ± SD (*n* = 3).

Stability and micellar structure of SMA-PS micelles were further investigated by fluorescence spectroscopy. When SMA-PS micelles were dissolved in 0.2 M phosphate buffer (pH 7.4) or in deionized water, the integrity of the micelle was conserved as seen by DLS with a discrete size distribution, where the fluorescence intensity of the SMA-PS micelle decreased extensively to only 20 to 28% as compared to free PS (Figure 5A,B). Furthermore, the stability of SMA-PS micelles was evaluated in the presence of detergents (SDS) (Figure 5A,B), urea that will break hydrogen bond (Figure 5C,D), and lecithin that is the major component of cell membrane (Figure 5E,F). The increase in fluorescence intensity with the increasing concentration of SDS, urea, and lecithin indicates disruption of micelles. The same effect was found when pH was higher than 8.5 (Figure 5C,D). 

### 3.6. Singlet Oxygen Generation from SMA-PS Micelles after Light Irradiation

#### 3.6.1. Electron Spin Resonance (ESR) Study: Prolongation of Singlet Oxygen Yielding Capacity in SMA-PS Micelles

**Figure 6:**
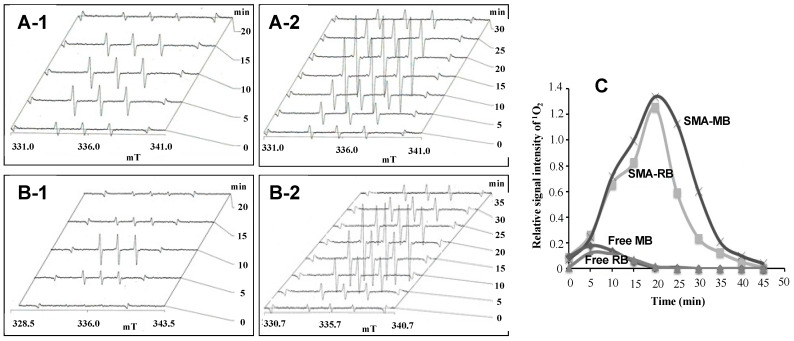
^1^O_2_ generation for free RB (**A-1**), cSMA-RB (**A-2**), free MB (**B-1**) and cSMA-MB (**B-2**) after light irradiation, as measured by electron spin resonance (ESR) spectroscopy. ESR measurement was carried out at the indicated time after the irradiation (see text for details). The relative ESR signal intensities of different PSs and changes over time are shown in (**C**).

^1^O_2_ generation form SMA-PS was then investigated by ESR, in which the signal intensity of ESR was increased upon irradiation of PS together with TMP solution. As shown in Figure 6, free PS reached the maximum ^1^O_2_ signal at 10 min of light irradiation, then started to decrease, and almost no signal was detected after 20 min light irradiation (Figure 6A-1,B-1,C. These results indicate that free PS could generate ^1^O_2_ only for a short time, which may suggest the decomposition of free PS. In contrast, both SMA-RB and SMA-MB micelles could generate ^1^O_2_ at much higher levels than free PS, and also for much longer periods of up to 30–40 min (Figure 6A-2,B-2,C. These results may indicate that PS in SMA micelles is more stable than free PS, which may be due to the existence of SMA to protect against the bleaching effect of light, and we thus did the following studies to validate this hypothesis.

#### 3.6.2. Bleaching Effect to Free PS and SMA-PS Micelles by Light Irradiation

**Figure 7:**
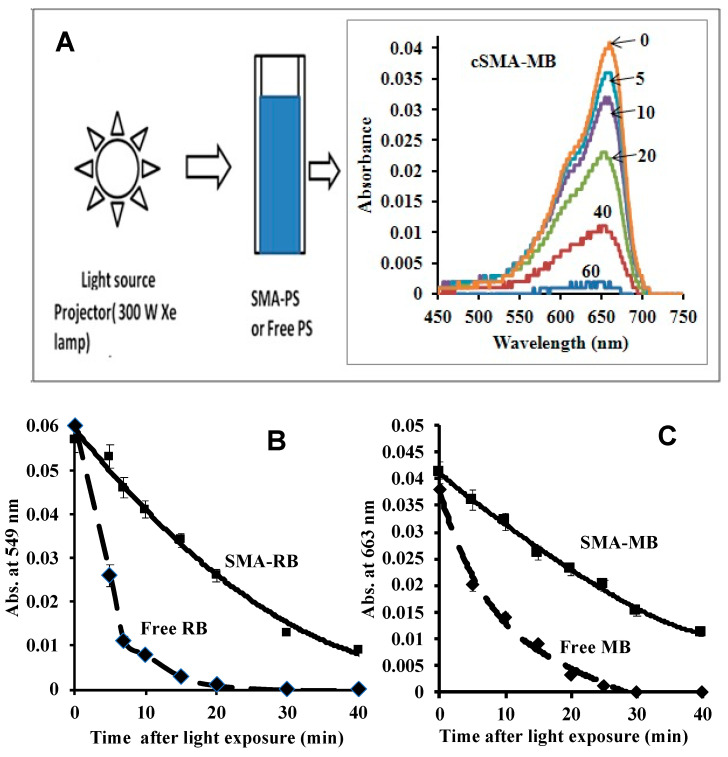
Photodegradation of SMA-PS micelles under irradiation of 12.9 mW using an Xe lamp 300 W (Kodak Ektagraphic III projector). UV absorption spectrum shows photodegradation of SMA-PS micelle (e.g., cSMA-MB) with time of light irradiation (**A**). The peak at 549.5 nm corresponding to RB (**B**) and 664.0 nm corresponding to MB (**C**) diminishes with respect to time of light irradiation. Data are mean ± SD (*n* = 3).

Free PS or SMA/PS was exposed to light irradiation for an indicated time, after which the UV/vis spectra were measured (Figure 7A). The UV/vis absorption spectra showed a significant decrease in absorption spectra after time-dependent light exposure (Figure 7A), which suggested the decomposition of PS. However, compared to free PS that exhibited almost complete decomposition after 20 min irradiation, SMA-PS micelles were decomposed much slower than free PS, of which about 20% remained after 40 min light irradiation (Figure 7B,C). These results are consistent to the results observed in Figure 6.

#### 3.6.3. Effect of Time Lagged TMP Addition on Singlet Oxygen Yielding Capacity of SMA-PS Micelles

**Figure 8:**
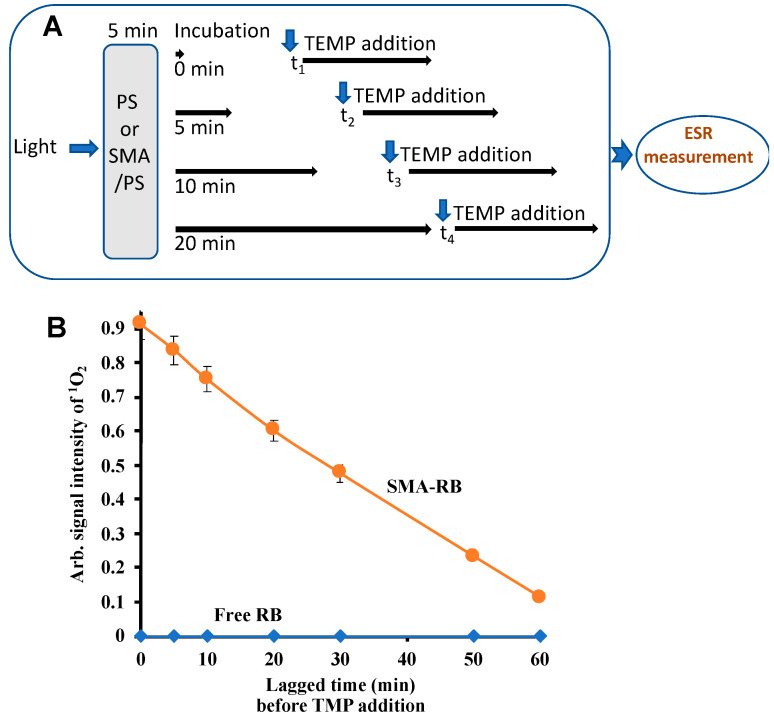
TMP/^1^O_2_ signal intensities against lagged time of incubation after light irradiation. (**A**) shows the diagrammatic representation of experimental condition of measuring ESR signals for SMA-PS micelles. The results of signal intensity of spin trapped ^1^O_2_ of cSMA-RB vs. free RB are shown in (**B**). Data are mean ± SD (*n* = 3). See text for details.

To clarify the stability of ^1^O_2_ in the presence of SMA-micelles, we further investigated through the experiment with lagged time addition of TMP, as represented in Figure 8A. Because the half life of ^1^O_2_ is very short (about 1.6 to 3.7 µs), addition of spin trap agent TMP after light irradiation did not result in any ESR signal in the case of free PS (Figure 8B). However, a relatively strong signal was detected in SMA-PS by the same experiment setting, and the signal decreased gradually depending on the time lags of TMP addition after light irradiation (Figure 8B). The results clearly indicated that SMA-PS micelles stabilize the ^1^O_2_, which may ensure the longer half-life of ^1^O_2_.

### 3.7. Intracellular Uptake of SMA-PS Micelle

**Figure 9:**
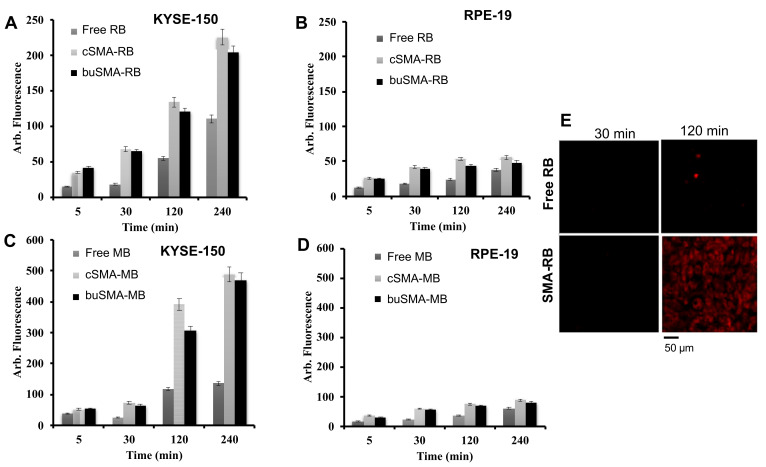
Intraellular uptake of SMA-RB micelles (**A**,**B**) and SMA-MB micelles (**C**,**D**) by cancer cells (KYSE-150) and normal cells (RPE-19). SMA-PSs were added into the culture media of KYSE-150 and RPE-19 cells; after the indicated time, the cells were collected and lysed with 10% TritonX-100^®^. The internalized SMA-PS was then quantified by fluorescence spectroscopy. (**E**) shows the interlization images of RB and SMA-RB in KYSE-150 cells by confocal laser microscope. Data are mean ± SD (*n* = 3). See text for details.

To investigate the cellular uptake properties of SMA-PS and free PS, human esophageal cancer KYSE-150 cells were used because esophageal cancer is one of the major target cancers of PDT in clinical settings, and normal cells (human retinal pigment epithelial cell RPE-19) were used for comparison. As seen in Figure 9A–D, the internalization of SMA-PS micelles and free PS were found to proceed in a time-dependent manner; however intracellular uptake in normal cells were much lower than that in cancer cells. More importantly, uptake of both SMA-PS micelles were significantly faster and higher than that of free PS, especially in tumor cells. The confocal laser microscopy images of KYSE-150 cells after RB and SMA-RB treatment also clearly showed the extensively rapid and high internalization of SMA-RB compared to free RB (Figure 9E). These results indicate the superior therapeutic potential of SMA-PS micelles to free PS.

### 3.8. In Vitro PDT Effect of SMA-PS

On the basis of the findings of prolonged and stable ^1^O_2_ generation, and superior intracellular uptake of SMA-PS compared to free PS as described above, we anticipated the improved PDT effect of SMA-PS, and thus investigated the PDT effect of SMA-PS in vitro.

**Figure 10:**
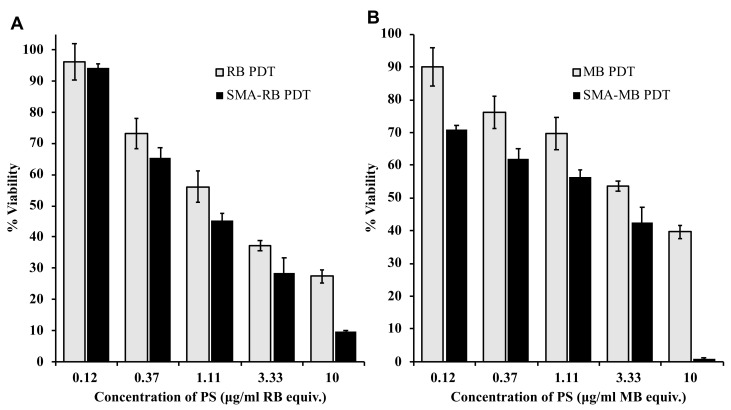
In vitro photocytotoxicity (PDT) of SMA-RB (**A**) and SMA-MB (**B**) in human esophageal cancer KYSE-150 cells. Cells (3000/well) were seeded in a 96-well plate; after overnight pre-incubation, indicated concentrations of SMA-PS were added. At 2 h after addition of SMA-PS, light irradiation was carried out by using a xenon light of 400–700 nm (21 J/cm^2^). After further 48 h incubation, the viability of cells was measured by MTT assay. Data are mean ± SD, *n* = 6~8. See text for details.

As shown in Figure 10, in cultured KYSE-150 esophageal cancer cells, dose-dependent photocytotoxicities of RB and WB after irradiation using a xenon light source (21 J/cm^2^) were observed, with the IC_50_ of 1.5 μg/mL and 3.6 μg/mL, respectively. Similarly, SMA-PS also showed photocytotoxicity in a dose-dependent manner; however, a more potent (2- to 3-fold) effect was found compared to free PS, i.e., the IC_50_ of PDT using SMA-RB and SMA-MB were 0.65 μg/mL and 1.6 μg/mL, respectively (Figure 10). These findings are in parallel with the results of intracellular uptake, as shown in Figure 9.

## 4. Discussion

We reported here SMA telomer micelles containing photosensitizers—SMA-RB and SMA-MB—which show more durable and controlled ^1^O_2_ generating capability than free PSs. All SMA-PS micelles prepared in this study were distributed in a discrete size range of 18.0 nm to 35.0 nm by DLS, and the molecular weight of micelles were in the range of 28 to 55 kDa in aqueous system, as observed by SLS and Sephadex chromatography with reasonable stability. The macromolecular nature of these micelles will offer another advantage in vivo by utilizing the EPR effect for tumor targeting; namely, highly facilitated accumulation into tumor tissues [11,13]. 

RB and MB are potential PSs that could be used in PDT, as well as in vivo imaging. Unlike many other PSs, for example photophyrin, laserphyrin, etc., these are non-toxic and their safety profiles are well-known. Namely, RB is widely used in medication and as a food additive; in addition, recently, MB was approved as an injective drug for methemoglobinemia [27,40,41]. However, these are low molecular weight compounds, which have short in vivo half-lives like other low molecular weight drugs; for example, it was known that RB was excreted rapidly by carrier-mediated hepatobiliary transport [41]. More importantly, these compounds do not have the properties to target tumors; namely, they cannot accumulate in tumor tissues selectively. In this context, the micelle formation using SMA as reported in the present study will render them a macromolecular nature with an apparent molecular size of >10 nm that will take advantage of the EPR effect to accumulate in tumor tissues. In addition, as SMA has the property to bind to albumin, which is abundant in circulation [38,39], SMA micelles of RB and MB exhibited albumin binding capacity (Figure 3, Table 2), resulting in the increased size of SMA-PS micelles to about 100 nm after binding to albumin (Table 2). This will further facilitate their in vivo behavior as macromolecules, consequently achieving successful EPR effect-based tumor selective accumulation. 

Regarding the release profiles of SMA micelles, SMA-RB showed a 4 to 4.5 times faster release rate than SMA-MB micelles (Figure 4A,B). This may be due to the higher ionic interaction of cationic MB than RB with poly-anionic SMA, in addition to the hydrophobic interaction in RB micelles. Furthermore, fluorescence quenching of SMA-PS in aqueous solution and physiological pH (Figure 5) indicated relatively tight interactions between PS and SMA, namely SMA-PS showed high stability in physiological conditions. Similar phenomena were also found in other SMA micelles prepared in our laboratory [22,34,35]. The fluorescence quenching of SMA-PS indicated the densely packed conformation of aromatic chromophore of PS showing π-π interaction, which resulted in dissipation of exited state energy, or efficient energy transfer to styrene residue in SMA micelles [22,34,35]; evidence of the embedded PS in the SMA polymer. The micelle formation of SMA-PS will ensure two steps of selectivity and safety for PDT. Namely, SMA-PS is stable in circulation as micelles with slow-release profiles (Figure 4), and its albumin binding property further ensures its stability (Figure 4E) and behavior as nano-drugs; whereas, after accumulation in tumor tissue by the EPR effect, it will be rapidly taken up by tumor cells (Figure 9). The micelles are expected to be disrupted upon cellular uptake as revealed by the increased fluorescence in the presence of lecithin, the major component of cell membrane (Figure 5E,F). Consequently, a strong PDT effect could be triggered selectively in the tumor by pinpointed irradiation.

To achieve high yield of ^1^O_2_ from PS, a number of parameters are important, such as solubility, quantum yield, stability, biocompatibility, fitting of absorption wavelength and wavelength of irradiating light source for excitation; whereas the laser beam has an inherent limitation. It is considered that the longer the wavelength, the better tissue penetration commences and thus irradiation wavelength selection is an important criterion for an effective PDT [7]. Another important aspect is the intensity of light. In this regard, xenon light is considered more useful compared to laser, which shows a much wider and continuous energy with a reasonable intensity. Thus, in this study, we used a xenon light source (109,000 LUX) covering an excitation wavelength for SMA-PS. When we use a xenon light source, we can choose a wide range of excitation wavelengths depending upon the requirement of each PS, whereas laser can emit only monochromatic or very narrow wavelengths, which may not fit to the maximal absorption of PS.

One important and interesting finding in this study is that SMA-PS can generate ^1^O_2_ for a much longer time, maintaining a reasonably higher intensity (Figure 6), thus making SMA micelles more advantageous photosensitizers than free PSs. The possible reasons behind this phenomenon are: (1) micelle formation will ensure stabilization of PS in the SMA micelles (Figure 7); and (2) ^1^O_2_ are entrapped in the hydrophobic environment of styrene rich milieu (Figure 8). It is known that solubility or affinity of ^1^O_2_ in hydrophobic solutions is 5 to 10 times more than in aqueous milieu. Thus, it could also be anticipated that ^1^O_2_ would exhibit high affinity to the polystyrene environment and may be well accommodated in the SMA micelles. This long-lived ^1^O_2_ generation from SMA-PS will thus show a much higher PDT effect than free PS, by readily reacting with targeted vital molecules in the tumor cells, causing tumor cell death.

Internalization of PS into tumor cells is a prerequisite for effective PDT because ^1^O_2_ and other radicals are highly reactive and unstable—they will quickly react with molecules nearby. Thus, intracellular production of ^1^O_2_ is an essential issue. Interestingly and importantly, in the present study, internalization of SMA-PS was much more rapid and higher than free PS (Figure 9). Moreover, intracellular uptake of SMA-PS in tumor cells was much higher than that in normal cells (Figure 9). It is well known that small molecules with Mw less than 1 kDa would diffuse through the plasma membrane or internalized by a receptor-mediated transporter protein, whereas macromolecules may enter into cells by pinocytosis or endocytosis [42]. RB and MB are hydrophilic molecules. Thus, these molecules are taken up mostly by receptor-mediated transporters which are largely influenced by the expression of transporter proteins. Moreover, endocytotic cellular uptake is believed to be more active in dividing cells such as tumor cells than non-dividing normal cells. Many macromolecular drugs including liposomes and polymer conjugates go through endocytotic pathways for their antitumor activity [42,43,44]; macromolecules, for example SMA-PS, will thus be extensively taken up by tumor cells, as evidenced in this study (Figure 9). However, the internalization of polymeric micelles is largely affected by the nature of the polymer used. For example, pegylated micelles are known to show much less internalization because the hydrated PEG-layer of the PEG micelles will interfere with the interaction with the receptor site on the cell surface, which is involved in endocytotic uptake of micelles, a known as the PEG-dilemma [45,46]. However, SMA-based micelles always show quick and active intracellular uptake, as evidenced in this study as well as in our previous studies [47]. This may be due to the hydrophobic nature of SMA which increases the affinity between SMA and surface receptors of the cells. The rapid and high intracellular uptake of SMA-PS will thus have advantages to be more effective than free PS for PDT.

Development of tumor environment responsive nano-drugs, e.g., increased release of active drugs under acidic tumor pH, have attracted much attention and is becoming an important principle for the design of nano-drugs [13,48]. In our laboratory, we have developed tumor environment (pH) responsive polymeric drugs, including SMA micelle drugs [38,48]; however, in this study, we did not find acidic pH responsive release profiles in SMA-PS (Figure 5). In this regard, however, it should be noted that the pH responsive nature is not the prerequisite for EPR effect-based tumor targeting—nano-sized drugs will accumulate in a tumor by the EPR effect as long as it is stable in circulation with a prolonged plasma half-life. In this context, stability in circulation is a critical issue for the EPR effect. However, tumor environment responsive release of active drugs and active intracellular uptake after accumulating in tumor tissues are the next critical and necessary steps beyond the EPR effect to achieve the satisfactory therapeutic effect. So, although SMA-PS did not show pH responsive release in this study, its stable micelle formation (Figure 2, Table 2), slow release in circulation and physiological conditions (Figure 4), as well as its albumin-binding property (Figure 3), will ensure targeted tumor accumulation by taking advantage of the EPR effect. Furthermore, the rapid intracellular uptake (Figure 9) and high ^1^O_2_ generation capacity (Figure 6) of SMA-PS will lead to highly effective PDT outcomes, which was partly confirmed by in vitro study using esophageal cancer cells (Figure 10).

In this study, we used human esophageal cancer cells to examine the biological activities of SMA-PS because esophageal cancer is currently a major target of PDT [5]. Compared to conventional surgery and endoscopic resection, PDT under endoscopy is a less invasive therapeutic option while having a wider medical indication, which recently has been recognized as a salvage treatment for esophageal squamous cell carcinoma after chemo-radiotherapy [5,49]. However, it is not widely applied worldwide, mostly due to the adverse side effects and complexity of perioperative management, e.g., high photosensitivity and long sunshade period [5], which are the disadvantages of low-molecular weight PSs used in clinical settings. In this context, nano-designed PS, for example SMA-PS as described in the current study, may largely overcome these problems by targeted delivery of PS into the tumor. The superior ^1^O_2_ generation capacity of polymeric micellar PS (i.e., SMA-PS) will further enhance the therapeutic effect, consequently resulting in much improved therapeutic outcome. We thus anticipate the clinical application of SMA-PS micelles, as well as other PS nano-formulations, for the treatment of esophageal cancer and other cancers in the near future.

## 5. Conclusions

Taken together, in this study we determined that SMA is an excellent micelle platform for RB and MB. Both SMA-RB and SMA-MB micelles are water-soluble, behave as macromolecules of 18 to 30 nm in size, and the micelles are relatively stable. Thus, these micelles are expected to accumulate in tumor lesions more selectively based on the EPR effect, thereby avoiding massive adverse effects. More importantly, these SMA micelles exhibit distinct advantages beyond EPR effect-based tumor accumulation, i.e., a prolonged and more potent ^1^O_2_ generation period, and faster uptake by tumor cells. Taken together, SMA-based macromolecular PSs for PDT offer a wide avenue for developing PSs with sustained ^1^O_2_ generation capacity for targeted tumor delivery and treatment, especially for endoscopic light accessible tumors. The high ^1^O_2_ generation capacity as evidenced with SMA-RB and SMA-MB may also be subject to some other SMA micelles, and even other polymers may also offer such exciting high ^1^O_2_ generation capacity similar to SMA; however, further investigations are warranted.

## Figures and Tables

**Table 1:** Properties and characterization of the Polystyrene-co-maleic acid-Photosensitizers (SMA-PSs) micelles.

SMA Micelles	^a^ SMA Mw.	^b^ Hydrodynamic Size (kDa)	^c^ Content (%W/W)	^d^ Surface Charge (ζ, mV) ^e^
cSMA-RB	1360	29	8.72	−43.03 ± 1.2
buSMA-RB	1581	34	9.41	−21.70 ± 0.8
cSMA-MB	1360	47	6.36	−46.03 ± 2.1
buSMA-MB	1581	52	5.41	−24.17 ± 0.5

^a^ Molecular weight of SMA copolymer was determined by using MALDI-TOF mass spectroscopy. ^b^ The hydrodynamic size of SMA-PS in aqueous medium was determined by Sephadex G-100 gel chromatography. ^c^ Content of PSs in SMA-PS micelles was measured using UV absorption spectroscopy with standard curves of free PS. ^d^ Surface charge on SMA-PS was determined by using Zeta potential and particle size analyzer, ELSZ-2 Otsuka, Japan. To measure the zeta potential, the sample solutions were dissolved in deionized water (1.5 mg/mL). ^e^ Data are mean ± SD (*n* = 3). cSMA-RB: carboxy Styrene-co-maleic acid-Rose Bangal micelle; buSMA-RB: butylated styrene-co-maleic acid-Rose Bengal micelle; cSMA-MB: carboxy Styrene-co-maleic acid-Methylene Blue micelle, buSMA-MB: butylated styrene-co-maleic acid- Methylene Blue micelle.

**Table 2:** Particle size (nm) of SMA-PS micelles in different aqueous milieu.

SMA Micelles	0.1 M Phosphate Buffer (pH 7.5)	Deionized Water	2% HSA in 0.1 M Phosphate Buffer (pH 7.5)
cSMA-RB	18.7 ± 1.1	35.4 ± 2.3	95.4 ± 11.3
buSMA-RB	23.5 ± 1.3	38.9 ± 1.2	107.4 ± 21.4
cSMA-MB	25.7 ± 1.6	41.3 ± 3.1	103.2 ± 13.5
buSMA-MB	27.5 ± 0.9	46.1 ± 2.7	111.8 ± 15.8

Particle sizes were measured by dynamic light scattering. Data are mean ± standard deviation (SD) (*n* = 3).

## Data Availability

The data presented in this study are available on request from the corresponding author.
